# An occupational mechanical job exposure index based on five Norwegian nationwide surveys of living conditions on work environment

**DOI:** 10.1186/s13104-024-06747-2

**Published:** 2024-03-28

**Authors:** Åsmund Hermansen

**Affiliations:** Faculty of Social Sciences, Department of Social Work, Child Welfare and Social Policy, OsloMet– Oslo Metropolitan University, Oslo, Norway

**Keywords:** Occupational mechanical job exposure index, Job exposure matrix, Register data, Working conditions

## Abstract

**Objectives:**

Nordic register data are not collected for research purposes, and various dimensions of working conditions are typically missing in register-based research. One way to address the issue of missing information on the work environment in register data is to utilize a job exposure matrix (JEM). The purpose of this paper is to document and provide access to a Mechanical Job Exposure Matrix (JEM) and a validated Occupational Mechanical Job Exposure Index based on the constructed JEM, allowing researchers to utilize the index in register-based research. The JEM and the Occupational Mechanical Job Exposure Index were created using data from five nationwide Surveys of Living Conditions on work environment conducted in Norway in 2006, 2009, 2013, 2016, and 2019, encompassing a total of 43,977 respondents. The index can be merged to register data using occupational codes (STYRK-98) and gender, which is information collected by the registries. The ultimate aim of constructing the index was to create a comprehensive measure of mechanical job exposures for use in future analyses of Norwegian register data.

**Data description:**

This paper provides the scripts documenting the construction of the Mechanical Job Exposure Matrix (JEM) and the Occupational Mechanical Job Exposure Index, as well as a data file including the matrix and the index. A script for merging the matrix and index to register data is also provided.

## Objective

The fact that Nordic registers often include the total population and longitudinal information over several decades makes them a ‘goldmine’ for research [1]. However, these data are not collected for research purposes, and various dimensions of working conditions are typically missing in register-based research. One way to address the issue of missing information on the work environment in register data is to utilize a job exposure matrix (JEM). JEMs have been developed for various specific exposures [2–5] and have been evaluated and applied in several European countries, including England [6], the Netherlands [7], Spain [8], Germany [9], France [10; 11], and also in overseas countries like the USA [12], Canada [13], and Australia [14]. Scholars in all four Nordic countries, Denmark, Finland, Norway, and Sweden, have also developed and evaluated national JEMs [4, 7, 15–17].

Based on data from five Norwegian nationwide Surveys of Living Conditions on work environment, Hermansen & Dahl [18] initially constructed a Mechanical Job Exposure Matrix (JEM). Subsequently, Hermansen & Dahl created and validated an Occupational Mechanical Job Exposure Index based on the constructed JEM. The five surveys used to create the JEM and the Occupational Mechanical Job Exposure Index were conducted in 2006, 2009, 2013, 2016, and 2019 and included a total of 43,977 respondents. The ultimate purpose of constructing the index was to create a summary measure of mechanical job exposures for use in future analyses of Norwegian register data. The purpose of this paper is to document and provide access to the Occupational Mechanical Job Exposure Index, allowing researchers to utilize the index in register-based research. The index comprises eight different mechanical job exposures. This paper also offers access to the necessary scripts documenting how these exposures were calculated and the completed Mechanical Job Exposure Matrix based on all five surveys. The scripts are in Stata-format. The index can be merged to register data using occupational codes (STYRK-98) and gender, which is information collected by the registries.

## Data description

The JEM is constructed based on data retrieved from the Norwegian nationwide Survey of Living Conditions on work environment performed in 2006, 2009, 2013, 2016 and 2019. All the surveys were conducted by Statistics Norway using Computer Aided Telephone Interviewing. The sample for each survey was randomly drawn from the population aged 18–69 years, which represented active working-age people in Norway. The highest response rate was 67.2% (2006) and the lowest response rate was 52.6% (2016).

The five surveys include eight questions mapping different types of mechanical job exposures: «Heavy lifting (> 20 kg) », «Hands above shoulder height», «Heavy physical work», «Neck flexion», «Squatting/kneeling», «Forward bending», «Awkward lifting» and «Standing/walking». Based on the individual response to these questions and using a cut-off value developed by an expert group in a Nordic project [19] and based on the scientific literature [20], individuals were identified as exposed versus non-exposed.

Whereas for the occupation-based exposures, the share of exposed individuals for each item within 268 JEM-groups was calculated, providing the overall share of exposed within a total of 323 unique occupational codes. This implies that occupational codes with a value of 0 on one of the exposures implies that none with these occupational codes, belonging to the same JEM-group, has provided an answer that involves exposure. In contrast, the value 100 means that all respondents with that occupational code, belonging to the same JEM-group, have provided an answer that involves exposure. For a detailed description regarding the construction of the Mechanical Job Exposure Matrix please see Hermansen & Dahl [18] p. 3–6.

A key of correspondence was developed, making it possible to the append the five surveys, converting the 4-digit STYRK-08 codes (used in 2013, 2016 and the 2019 survey) into 4-digit STYRK-98 codes (used in the 2006 and 2009 survey). The STYRK-98 codes are based on ISCO-88 (COM). When faced with the choice of having more than one STYRK-98 code to select, the STYRK-98 code with the highest N in the 2006 and 2009 survey combined was selected. The chosen key of correspondence led to changes in 28% of the 4-digit STYRK-08 occupational codes, thus 72% remained unchanged.

Table 1 below includes the scripts documenting the construction of the Mechanical Job Exposure Matrix and the Occupational Mechanical Job Exposure Index (Data file 1), as well as a script containing the key of correspondence converting the 4-digit STYRK-08 codes into 4-digit STYRK-98 codes and the procedure for merging the index to register data (Data file 2). The complete Mechanical Job Exposure Matrix and the Occupational Mechanical Job Exposure Index are included in Data file 3 “NSD3131.dta”. Data file 4 contains a complete codebook and Data file 5 is the validation study performed by Hermansen & Dahl [18] published in BMC Public Health.

**Table 1:** Overview of data files

Label	Name of data file/data set	File types (file extension)	Data repository and identifier (DOI or accession number)
*Data file 1*	*Constructing_Mechanical_exposures_index.do*	*Stata script file (.do)*	*Sikt–Norwegian Agency for Shared Services in Education and Research* *(*10.18712/NSD-NSD3131-V2*)* [21]
*Data file 2*	*Merging_Mechanical_exposures_index.do*	*Stata script file (.do)*	*Sikt–Norwegian Agency for Shared Services in Education and Research* *(*10.18712/NSD-NSD3131-V2*)* [21]
*Data file 3*	*NSD3131.dta*	*Stata data file (.dta)*	*Sikt–Norwegian Agency for Shared Services in Education and Research* *(*10.18712/NSD-NSD3131-V2*)* [21]
*Data file 4*	*NSD3131 codebook.html*	*HyperText Markup Language (.html)*	*Sikt–Norwegian Agency for Shared Services in Education and Research* *(*10.18712/NSD-NSD3131-V2*)* [21]
*Data file 5*	*Hermansen & Dahl (2022)*	*Portable document format (.pdf)*	*Sikt–Norwegian Agency for Shared Services in Education and Research* *(*10.18712/NSD-NSD3131-V2*)* [21]

## Limitations

- The matrix and the Occupational Mechanical Job Exposure Index is developed for the use in analysis based on Norwegian register data, which introduces a limitation on its broader applicability.
- There is an absence of an official key of correspondence between the 4-digit STYRK-98 and the 4-digit STYRK-08 codes. This lack of an established mapping between the two code systems has been confirmed through correspondence with Statistics Norway, specifically their section for labour market statistics. As a result, a key of correspondence had to be developed solely for the creation of the Job Exposure Matrix.
- The absence of an official correspondence key poses a potential challenge when attempting to compare or reconcile data between the two code systems. This discrepancy might introduce errors or inconsistencies when trying to align or merge datasets that utilize different versions of the STYRK classification system.

## Data Availability

The data described in this Data note can be freely and openly accessed through Sikt (Norwegian Agency for Shared Services in Education and Research) under 10.18712/NSD-NSD3131-V2. Please see Table 1 and reference [21] for details and links to the files and data. Note that Sikt (Norwegian Agency for Shared Services in Education and Research) requires users to login before accessing the data. Please also note that Data File 1, 2, and 5 are to be found under “Study documentation” and “Summary” with the heading “Other materials” in the Sikt repository.

## Abbreviations

JEM: Job Exposure Matrix
STYRK: Norwegian Occupational Catalogue
STYRK-98: Standard for occupational classification used from 1998
STYRK-08: Standard for occupational classification used from 2008
